# Treatment Effect and Drug-Resistant Mutations in Chinese AIDS Patients Switching to Second-Line Antiretroviral Therapy

**DOI:** 10.1371/journal.pone.0110259

**Published:** 2014-10-17

**Authors:** Min Zhang, Mingquan Shang, Weiwei Yang, Junli Chen, Zhe Wang, Hong Shang

**Affiliations:** 1 Key Laboratory of AIDS Immunology of National Health and Family Planning Commission, Department of Laboratory Medicine, The First Affiliated Hospital, China Medical University, Shenyang, China; 2 Collaborative Innovation Center for Diagnosis and Treatment of Infectious Diseases, Hangzhou, China; 3 Disease Prevention and Control Center of Henan Province, Zhengzhou, China; University of Rochester, United States of America

## Abstract

This study aimed to investigate treatment effect, drug resistance changes, and their influencing factors in Chinese AIDS patients after switching to second-line antiretroviral therapy, and thus provide important information for the scale-up of second-line antiretroviral treatment in China. In Weishi county of Henan province, where second-line antiretroviral therapy was introduced early in China, 195 AIDS patients were enrolled, of which 127 patients met the switching criterion and 68 patients volunteered to switch drugs without meeting the switching criterion. CD4 cell count, viral load and in-house PCR genotyping for drug resistance were measured for all 195 subjects before drug switch, as well as 6 and 12 months after drug switch. Extensive secondary mutations to the protease inhibitor were observed, which suggested that long-term drug resistance surveillance is necessary for patients switching to second-line antiretroviral therapy. Multidrug resistance and cross-resistance were extensive in Chinese patients that experienced first-line treatment failure. Patients need timely CD4 count, viral load, and drug resistance monitoring in order to switch to second-line therapy under conditions of relatively good immunity and low viral duplication levels.

## Introduction

Highly active antiretroviral therapy (HAART) is currently the most effective therapy for the treatment of acquired immune deficiency syndrome (AIDS) [1]. However, the characteristic high mutation level of human immunodeficiency virus (HIV), along with selection pressure of antiretroviral drugs, usually cause rapid occurrence of drug resistance of HIV, which has been the main reason for clinical failure of antiretroviral treatment. In America and Europe, drug resistance of HIV existed in more than 60% of patients who were antiretroviral treatment failure [2].

China initiated a large-scale program for free antiretroviral treatment in 2003, with zidovudine (AZT)/stavudine (D4T) + didanosine (DDI) + nevirapine (NVP) as the main first-line therapy regimen. By October 2011, more than 130,000 patients with AIDS had received HAART. The increase in availability and access to free antiretroviral treatment had resulted in considerably prolonged survival time, improved quality of life and decreased mortality rate in AIDS patients [3]. But with the increase of HAART treatment duration, treatment failure and drug resistance occurred in more and more patients. In a cross-sectional survey conducted in 2007, Min Zhang et al. [4] studied HIV infected patients living in 7 provinces in China and reported that the rate of drug resistance was significantly higher in patients receiving first-line treatment for more than 6 months than that in patients receiving treatment for less than 6 months. In 2008, Min Zhang [5] conducted a cohort study on HIV infected patients living in Weishi county of Hunan province of China. The results showed that, among 88 patients who had received the first-line antiretroviral treatment, only 1% of patients had drug resistance before treatment. The rate increased to 15% after the patients received treatment for 12 months, and increased to 17% after 36 months. As the increasing rate of drug resistance resulted in diminishing efficacy of the free antiretroviral treatment, in 2009 China initiated the switch to second-line antiretroviral therapy for patients with failure of first-line treatment.

Some foreign researchers reported that extensive drug-resistant mutations were observed before switching therapy regimens. Regarding treatment results after switching to second-line antiretroviral regimens, there is variance among reports from different regions and countries [6]–[8]. In China, with the expanding of the free antiretroviral treatment and the extending of treatment duration, the rate of drug resistance increases over time. What would be the treatment results after patients switch to second-line antiretroviral therapy? Would accumulation of drug-resistant mutations during the first-line treatment have an impact on the second-line treatment results? Would new drug-resistant mutations appear soon after the administration of second-line regimens? In China, no study has examined efficacy and drug resistance after patients switch to second-line regimens. In Weishi county of Henan province, where antiretroviral treatment started early, more than 2,000 HIV infected individuals had received antiretroviral treatment, and rates of treatment failure and drug resistance were increasing annually [5]. From August 2009, some patients began to switch to second-line therapy. We conducted a prospective observational cohort follow-up study on these AIDS patients in Weishi county who switched to second-line therapy.

## Materials and Methods

### Ethics statement

This study was approved by the Institutional Review Board of the First Affiliated Hospital of China Medical University (Shen Yang, China). All participants ≥18 years of age, written informed consent was obtained from all subjects.

### Study subjects

Our research is an observational study; we did not determine or influence the course of treatment. We just collected the therapy information and blood samples of patients in the second-line antiretroviral therapy pilots in Henan province.

One hundred and ninety-five AIDS patients living in Weishi county of Henan province were enrolled in August 2009 and February 2010, respectively. In accordance with Chinese National Free AIDS Antiretroviral Therapy Manual issued in 2009 [9] and WHO antiretroviral therapy for HIV infection in adults and adolescents [10], virological failure defines as plasma HIV-1 RNA level >400 copies/mL after 6 months of treatment or HIV-1 RNA level >1000 copies/mL after initial virological suppression. Virological failure of first-line therapy was the main reason to switch to second-line treatment. 127 patients were first-line virological failure, after first-line virological failure happened, the patients continued the old treatment for one month with rectified treatment compliance. After that, if the viral load of the patient was higher than 1000 copies/mL, the patient switched to second-line therapy. As a result, viral loads of all the 127 subjects were higher than 1000 copies/mL. The remaining 68 patients who were virological suppression after first-line therapy, but they requested to switch to the second-line drugs due to significant toxicity and side effects of their first-line drugs.

The second-line antiretroviral regimen used was Lamivudine (3TC) + tenofovir (TDF) and lopinavir + ritonavir (LPV/r), as recommended by WHO [9]. Patients were followed up and their CD4, viral loads and drug resistance were measured 6 and 12 months after starting second-line treatment. Peripheral venous blood was obtained from each patient using an EDTA-3 K tube containing anticoagulants. CD4+T lymphocytes were counted within 24 hours. Blood for measurement of viral loads and drug resistance was centrifuged for 15 minutes, and plasma separated from blood was stored at −80°C.

### CD4+T lymphocyte count

Absolute CD4+, CD8+, and CD3+T lymphocytes were counted with the FACSCalibur flow cytometer (Becton Dickinson, USA).

### Viral load measurement

Viral loads were measured using COBAS AmpliPrep/COBAS TaqMan HIV-1 Test (Roche, Switzerland).

### Drug-resistant mutation analysis

HIV-1 viral RNA was extracted from 140 ul plasma with the QIAamp Viral RNA Mini Kit (Qiagen Inc, USA). Reverse transcription-polymerase chain reaction (RT-PCR) was carried out with the One-step RNA PCR Kit (AMV) within 4 hours, and 1300 bp of reverse transcriptase (RT) and protease (PR) genes of HIV-1 were amplified with nested PCR, including the whole PR and the first 300 amino acids of RT [5]. The PCR product was then analyzed with ABI 3130 Genetic Analyzers (ABI, USA) using the chain termination method.

### Sequence analysis and statistical analysis

Sequencher 4.10.1, NTI advance 9 and ContigExpress were used for sequence editing, assembly and rectification. The resulting sequences were compared with the Stanford University HIV drug resistance database (http://hivdb.stanford.edu) version 6.1.1. for analysis of HIV drug-resistant mutations and drug resistance. Statistical Package for Social Sciences (SPSS) version 15.0 was used for statistical analysis; data are shown as means ± standard deviation. Differences between groups were compared with chi-square tests and Fisher exact probability tests. Logistic regression analysis was used to identify the influencing factors of viral inhibition failure (viral load <400 copies/mL) 12 months after switching to second-line treatment, and odds ratios (OR) and confidence intervals (CI) were used to express the relative strength of association. A P value <0.05 was considered statistically significant. Viral loads <400 copies/mL after treatment were considered to have virological inhibition. The average viral load was expressed with the logarithmic (log_10_) form.

## Results and Discussion

### General information and drug resistance of patients before drug switches

Of 127 patients who met the second-line treatment criterion, 71 patients (61.5%) were male and 56 patients (38.5%) were female. The average age was 45 (32–60) years. Among these 127 patients, 88 patients (69.3%) were drug resistant, 108 patients (85%) had CD4 cell counts <350 cells /mm^3^, and all patients had viral loads >1,000 copies/mL (Table 1). Before drug switches, the average first-line treatment duration of 127 patients was 54.8 (13–73) months. The first-line regimens used included AZT+DDI+NVP (70.0%), AZT+DDI+3TC (4.3%), AZT+3TC+NVP (8.6%), D4 T+DDI+NVP (8.6%) and others (8.5%).

**Table 1:** General information of subjects before drug switch.

		Patients meeting drugswitching criterion(127 patients)	Percent	Patients failing to meetDrug switching criterion(68 patients)	Percent
Gender	Male	71	61.50%	42	61.80%
	Female	56	38.50%	26	38.20%
CD4 cell count (cells /µl)	<200	72	56.70%	22	32.40%
	200–350	36	28.30%	20	29.40%
	≥350	19	15.00%	26	37.20%
viral loads (copies/mL)	>1,000	127	100%	0	0
Drug resistance	NRTI	69	54.30%	5	7.40%
	NNRTI	88	69.30%	7	10.30%
No. of sequence		122	96.10%	8	11.80%

Of 68 patients who did not meet the second-line treatment criterion, 42 patients (61.8%) were male and 26 patients (38.2%) were female. The average age was 44 (33–59) years. Among these 68 patients, 7 patients (10.3%) were drug resistant, 42 patients (61.8%) had CD4 cell counts <350 cells /mm^3^, and all patients had viral loads <1,000 copies/mL (Table 1). Before drug switches, the average first-line treatment duration of 68 patients was 50.8 (24–70) months. The first-line regimens used included AZT+DDI+NVP (67.6%), AZT+DDI+3TC (6.0%), AZT+3TC+NVP (8.8%), D4 T+DDI+NVP (8.8%) and others (8.8%).

### Treatment results after drug switches

All patients were 95% or more adherent to therapy after switching to second-line therapy according to the questionnaire results. One hundred and fifty-six patients were followed up 6 months after switching to second-line therapy and one hundred and sixty-seven patients were followed up at 12 months.

Of 127 patients who met the second-line treatment criterion, 98 and 113 patients were followed up at 6 and 12 months. At 6 months, 29 subjects were loss-of-follow-ups during the study, 15 of these patients were migrant workers, when we went to collect the blood samples and therapy information they were away from Weishi county of Henan province, but according to the drug receiving records in CDC, they continued second-line therapy; The remaining 14 patients, we totally can not contact with them. At 12 months, we got the 15 migrant workers’ blood samples and therapy information, but the 14 patients who we did not contact with at 6 months were still lost contact. After switching to second-line treatment, the CD4 average increased to 220.0±137.2 at 6 months with an increase of 16.6 cells/µl, and 280.0±160.0 (P<0.01) at 12 months with an increase of 76.6 cells/µl, showing a gradual increase trend. Percentage of patients with CD4 <350 cells/µl reduced to 70% (79/113) at 12 months. At 6 months, the percentage of patients with viral loads <400 copies/mL increased from 0% before drug switches to 60.8%, and the viral load average VL(log_10_) decreased from 4.6±0.8 before drug switches to 2.3±1.7; at 12 months, the percentage of patients with viral loads <400 copies/mL increased to 67.3%, and the viral load average VL (log_10_) decreased to 2.0±1.7 (Table 2) (P<0.01).

**Table 2:** CD4 count, viral load and viral inhibition rates before, 6 months after, and 12 months after drug switch.

	CD4 count		Viral load (lg)		Viral inhibition	
Treatment duration	Patientsmeetingswitchingcriteria	Patients failingto meet switchingcriteria	Patientsmeetingswitchingcriteria	Patients failingto meetingswitching criteria	Patientsmeetswitchingcriteria	Patients failingto meetswitchingcriteria
Before drugswitch	203.4±153.0	261.0±127.0	4.8±0.8	1.2±0.6	0	89.7%
At 6months	220.0±137.2	322.2±164.8	2.3±1.7	1.6±1.4	60.8%	83.1%
At 12months	280.0±160.0	329.4±134.0	2.0±1.7	1.3±1.1	67.3%	90.7%

Of 68 patients who volunteered for second-line treatment, 58 and 54 patients were followed up at 6 and 12 months. The 14 loss-of-follow-up patients were migrant workers, they were away from Weishi county of Henan province that we can not contact with them. After switching to second-line treatment, the CD4 average was 322.2±164.8 at 6 months with an increase of 61.2 cells/µl, and 329.4±134.0 (P>0.05) at 12 months with an increase of 68.4 cells/µl, showing insignificant difference compared to that before drug switches. The percentage of patients with viral loads <400 copies/mL decreased from 89.7% before drug switches to 83.1% at 6 months, and increased to 90.7% at 12 months (P>0.05); the viral load average VL (log_10_) increased from 1.2±0.6 before drug switches to 1.6±1.4 at 6 months, and then decreased to 1.3±1.1 at 12 months (Table 2) (P>0.05).

### Drug resistance in patients after drug switches

Of 127 patients, the rate of drug resistance was 69.3% (88/127) before second-line treatment, 23.5% (23/98) and 8.8% (10/113) after 6 and 12 months after drug switches, showing a significant decrease (P<0.001) compared to that before drug switches.

Of 68 patients who volunteered to participate in second-line treatment, drug-resistant mutations existed in 7 patients before drug switches. At 6 months, 58 patients were followed-up and 4 patients were found to have drug resistance, of which 2 patients were new to carry drug resistance. At 12 months, 54 patients were followed-up and 1 new patient was found to have drug resistance. All drug resistance found were high drug resistance to nonnucleoside reverse transcriptase inhibitor (NNRTI). No significant difference was observed between drug resistance before and after second-line treatment (P>0.05).

### Cross-resistance and multidrug resistance before and after drug switches

Before second-line treatment, rates of cross-resistance and multidrug resistance were high. The rates of drug resistance to NRTI and cross-resistance to AZT/D4 T/DDI were 54.3% (69/127) and 51.2% (65/127), respectively. Rates of drug resistance to NRTI significantly decreased to 17.3% (17/98) and 7.1% (8/113) at 6 and 12 months (P<0.001) after second-line drug switches; Rates of cross-resistance to AZT/D4 T/DDI significantly decreased to 16.3% (16/98) and 7.1% (8/113) at 6 and 12 months (P<0.001), respectively. Before drug switches, the rate of drug resistance to NNRTI was 69.3% (88/127), it decreased to 23.5% (23/98) and 11.5% (13/113) at 6 and 12 months after drug switches, respectively. The multidrug resistance rate to NRTI and NNRTI decreased from 54.3% (69/127) to 7.1% (8/113) after one year of second-line treatment (Table 3).

**Table 3:** Drug resistance analysis before and after second-line treatment.

	NRTI		NNRTI		NRTI and NNRTI	
	Drugresistance	High drugresistance*	Drugresistance	High drugresistance	Drugresistance	High drugresistance
Before drugswitch(N = 127)	54.3% (69)	42.5% (54)	69.3% (88)	66.1% (84)	54.3% (69)	42.5% (54)
At 6 months(N = 98)	17.3% (17)	14.3% (14)	23.5% (23)	23.5% (23)	17.3% (17)	14.3% (14)
At 12 months(N = 113)	7.1% (8)	4.4% (5)	11.5% (13)	11.5% (13)	7.1% (8)	4.4% (5)

*A score >60 get from Stanford University drug resistance database was regarded as high drug resistance.

### Drug resistance to 3TC and TDF before and after drug switches

3TC and TDF, which were included in second-line therapy regimens, are NRTI. Rates of drug resistance to 3TC and TDF before drug switches were 30.7% (39/127) and 45.7% (58/127), respectively. After 6 and 12 months of second-line treatment, rates of drug resistance to 3TC decreased to 11.2% (11/98) and 2.7% (3/113) respectively, and rates of drug resistance to TDF decreased to 15.3% (15/98) and 6.2% (7/113) respectively (Table 4). At 6 and 12 months, rates of cross-resistance to 3TC and TDF also decreased from 28.3% (36/128) before second-line treatment to 9.2% (9/98) and 1.8% (2/113), respectively (Table 4). In which, there were 3 cases of new cross-resistance at 6 months, and 1 new case at 12 months. At 12 months, 86.1% (31/36) of patients who showed cross-resistance to 3TC and TDF had a HIV RNA <400 copies/mL.

**Table 4:** Resistance to 3TC and TDF before and after drug switch.

	3TC	TDF	3TC & TDF
Before drug switches (N = 127)	30.7% (39)	45.7% (58)	28.3% (36)
At 6 months (N = 98)	11.2% (11)	15.3% (15)	9.2% (9)
At 12 months(N = 113)	2.7% (3)	6.2% (7)	1.8% (2)

### Analysis of associated factors of treatment results

All patients were 95% or more adherent to therapy 6 and 12 months after second-line treatment initiation. At 12 months, viral loads in 32.7% (37/113) of patients were not inhibited under 400 copies/mL. Results of univariate analysis showed that HIV-1 RNA >100,000 copies/mL before drug switches correlated with ineffective inhibition of viral loads after regimen change, the OR values was 3.95 (95% CI: 1.20, 13.0) (P<0.05). The OR of CD4<100 cells/µl was 5.14 (95% CI: 0.99, 26.71), however, the P value were insignificance statistical (P>0.05) (Table 5). The OR values of virological inhibition failure in female patients and 45 years old patients were 1.19 (95% CI: 0.34, 4.18) (P>0.05) and 2.11 (95% CI: 0.64, 6.95) (P>0.05) respectively. The second-line regimen with only 1 NRTI drug changed compared to the first-line regimen, OR = 1.31 (95% CI: 0.13, 13.74) (P>0.05). The OR of more than 3 years of first-line antiretroviral treatment duration was 1.01 (95% CI: 0.46, 1.68) (P>0.05) (Table 5).

**Table 5:** Associated factors of virological inhibition failure 12 months after second-line treatment initiation with logistic regression analysis.

Associated factors		OR (95% CI)
Female		1.19 (0.34, 4.18)
>45 years old		2.11 (0.64, 6.95)
CD4 cell count before second-line treatment	<100 cells/mL	5.14 (0.99, 26.71)
	100–200 cells/mL	1.72 (0.35, 8.48)
	≥200 cells/mL	1.0
Viral loads before second-line treatment	1000–10000 copies/mL	1.0
	10000–100000copies/mL	0.87 (0.24, 3.08)
	≥100000 copies/mL	3.95 (1.20, 13.0)*
No. of NRTI drugs changed compared to the first-line regimen	1 NRTI drug	1.31 (0.13, 13.74)
	2 NRTI drugs	0.76 (0.07, 7.98)
Treatment duration of first-line therapy	<3 years	1.0
	≥3 years	1.01 (0.46, 1.68)

*P<0.05, which were considered statistical significance.

In this study, 127 patients with first-line antiretroviral treatment failure switched to the second-line regimen after an average of 54.8 months of first-line treatment. Before second-line treatment, viral loads in all patients were higher than 1000 copies/mL. The patients had low immunity, 85% of which had a CD4 count lower than 350 cells/µl, and 69.3% harbored drug resistance. After switching to second-line regimen (3TC+TDF+LPV/r) for 12 months, 67.3% of patients had effective viral inhibition; the average of CD4 cell count had an increase of 76.6 cells/µl; the rate of CD4<350 cells/µl decreased to 70%; and the rate of drug resistance decreased to 8.8%. The significant improvement indicated that switching to second-line antiretroviral drugs led to good treatment effect.

Chinese second-line antiretroviral therapy is the first treatment choice in Europe and America, which produced satisfactory results in antiretroviral-naïve patients with AIDS [8], [11]. While in most developing countries, second-line antiretroviral therapy is a substitute when first-line treatment fails. Extensive drug-resistant mutations accumulate in bodies of patients who experienced first-line treatment failure. MC Hosseinipour et al. [7] reported that 85.2% of 88 patients had viral loads lower than 400 copied/mL after one year treatment with (AZT+3TC+TDF+LPV/r) second-line therapy regimen, despite the extensive drug resistance at baseline. In a large multicenter study of 632 patients receiving second-line therapy for 16.6 months in Africa and Asia, only 54% of patients had effective inhibition of virus loads [8]. Possible reasons for significantly different efficacy of second-line therapy are accumulation of drug resistance before drug change or poor adherence to therapy. In this study, 67.3% inhibition rate of viral load was achieved 12 months after drug change, despite the 69.3% in drug-resistant mutation before second-line therapy. As second-line antiretroviral drugs are relatively costly, in addition to the timely switch to second-line drugs after first-line treatment failure, it is essential to strengthen the education of treatment adherence for patients to fully utilize the limited drug sources and obtain good treatment effect.

In our study, before switching to second-line antiretroviral therapy, drug resistance existed in 69.3% of patients who met drug switching criteria. Cross-resistance and multidrug resistance also existed severely. The rates of cross-resistance and multidrug resistance to NRTI and NNRTI were 69.3% and 54.3%, respectively. However, after one year of second-line treatment, the overall drug resistance rate decreased to 8.8%, and rates of cross-resistance and multidrug resistance also decreased to 11.5% and 7.1%. Our results showed that, although severe cross-resistance and multidrug resistance existed after first-line treatment failure, second-line therapy effectively decreased the rate of drug resistance, as well as the epidemic risk of drug-resistant bacterial strains. Analysis of cross-resistance to NRTI (3TC and TDF), the main drug in the second-line antiretroviral regimen, showed that before drug change, 28.3% of patients had cross-resistance to 3TC and TDF, along with viral loads >1000 copies/mL. After 12 months of second-line treatment, viral loads in 86.1% of patients were effectively inhibited. In these patients, although there was only LPV/r playing a role, satisfying effect were observed and the cross-resistance rate to the 3TC and TDF decreased to 1.8%. It was noticeable that in our study, although drug resistance to the protease inhibitor (PI) was not found, extensive PI secondary drug-resistant mutations were detected. In case PIs primary drug-resistant mutations occur, it will lead to high PI drug resistance. Considering that there were new patients found to have drug-resistant mutations at 6 and 12 months, drug resistance surveillance should be continued to observe the cross-resistance, multidrug resistance and PI drug-resistant mutations after long-term second-line treatment.

Among patients meeting drug switching criteria, we performed a regression analysis of those patients whose virus loads were not inhibited under 400 copies/mL after drug change. The results showed that viral load higher than 100,000 copies/mL before drug change was a risk factor of virological inhibition failure. Similarly, a European study demonstrated that after switching to second-line antiretroviral regimen (including the protease inhibitor); levels of viral loads were more easily inhibited in patients with lower viral loads [8]. Similar findings have been reported that factor associated with HIV-1 RNA >400 copies/mL at 12 months on univariate analysis included having a presenting CD4 count <50 cells/µl and HIV-1 RNA >100,000 copies/mL [7]. However, in our research, lower CD4 count was not a significant factor associated with virological failure. This may be affected by the loss-of-follow-up subjects. In our study, 68 patients did not meet drug switching criteria and volunteered to change drugs. Before second-line treatment, the CD4 count average was 261.0 cells/µl, the rate of viral loads <400 copies/mL was 89.7%, and there were 7 cases of drug resistance; after 12 months of second-line treatment, the CD4 count average was 329.4 cells/µl, the rate of viral load inhibition was 90.7%, and there was 1 case of drug resistance. Although after second-line treatment, no significant differences were observed in virological immunological performance compared to that before drug change, ability of immunity and viral inhibition were still maintained in relatively high levels. These results suggested that in order to fully and effectively utilize the limited second-line drug sources and obtain good clinical effect, it is important to increase the measuring viral loads of treated patients.

## Conclusions

Multidrug resistance and cross-resistance existed severely in Chinese patients that experienced first-line treatment failure. These patients need to be monitored timely on viral loads and drug resistance and switch to second-line therapy under conditions of relatively low viral duplication levels. Satisfactory results were observed in patients treated with Chinese second-line antiretroviral therapy regimen (3TC+TDF+LPV/r) for 12 months, with no drug resistance to PI found. However, extensive secondary mutations to PI were observed, which suggested that the long-term drug resistance surveillance is necessary for patients switching to second-line antiretroviral therapy.
